# Genetic diversity and antibody responses against *Plasmodium falciparum* vaccine candidate genes from Chhattisgarh, Central India: Implication for vaccine development

**DOI:** 10.1371/journal.pone.0182674

**Published:** 2017-08-07

**Authors:** Priyanka Patel, Praveen K. Bharti, Devendra Bansal, Rajive K. Raman, Pradyumna K. Mohapatra, Rakesh Sehgal, Jagadish Mahanta, Ali A. Sultan, Neeru Singh

**Affiliations:** 1 National Institute for Research in Tribal Health, Indian Council of Medical Research, Garha, Jabalpur, Madhya Pradesh, India; 2 Department of Microbiology and Immunology, Weill Cornell Medicine - Qatar, Cornell University, Qatar Foundation - Education City, Doha, Qatar; 3 Community Health Centre Janakpur, District Baikunthpur, Chhattisgarh, India; 4 Regional Medical Research Centre, NE, Indian Council of Medical Research, Dibrugarh, Assam, India; 5 Department of Parasitology, Postgraduate Institute of Medical Education and Research, Chandigarh, Punjab, India; London School of Hygiene and Tropical Medicine, UNITED KINGDOM

## Abstract

The genetic diversity in *Plasmodium falciparum* antigens is a major hurdle in developing an effective malaria vaccine. Protective efficacy of the vaccine is dependent on the polymorphic alleles of the vaccine candidate antigens. Therefore, we investigated the genetic diversity of the potential vaccine candidate antigens i.e. *msp-1*, *msp-2*, *glurp*, *csp and pfs25* from field isolates of *P*.*falciparum* and determined the natural immune response against the synthetic peptide of these antigens. Genotyping was performed using Sanger method and size of alleles, multiplicity of infection, heterogeneity and recombination rate were analyzed. Asexual stage antigens were highly polymorphic with 55 and 50 unique alleles in *msp-1* and *msp-2* genes, respectively. The MOI for *msp-1* and *msp-2* were 1.67 and 1.28 respectively. A total 59 genotype was found in *glurp* gene with 8 types of amino acid repeats in the conserved part of RII repeat region. The number of NANP repeats from 40 to 44 was found among 55% samples in *csp* gene while *pfs25* was found almost conserved with only two amino acid substitution site. The level of genetic diversity in the present study population was very similar to that from Asian countries. A higher IgG response was found in the B-cell epitopes of msp-1 and csp antigens and higher level of antibodies against csp B-cell epitope and glurp antigen were recorded with increasing age groups. Significantly, higher positive responses were observed in the csp antigen among the samples with ≥42 NANP repeats. The present finding showed extensive diversity in the asexual stage antigens.

## Introduction

Globally about 3.2 billion people are at risk of malaria and 214 million new malaria cases and 438,000 deaths was reported in 2015[1]. South East Asia Region contributed 10% of the global malaria cases, of which India alone accounts for 70% of the cases [1]. *Plasmodium falciparum* contributes 67% of the total malaria cases in India with a greatly varied proportion from 0% to 93% in different states [2,3]. Malaria is a major health problem in rural/tribal areas of the Central Eastern and North Eastern States of India, which are having large groups of ethnic population [4]. Currently, an increasing number of countries including India are in the process of eliminating malaria. However in India, despite of scaling up of interventions such as use of insecticides treated net (ITN), indoor residual spray (IRS), improved diagnostic test and treatment using artemisnin combination therapy (ACT), malaria positivity is increasing 0.78% to 0.95% from 2013 to 2015 [2].

Therefore, highly effective malaria vaccine is definitely needed to achieve the target of malaria elimination. Although there is no effective vaccine as on date, however a number of potential stage specific vaccine candidate antigens of *P*. *falciparum* are under various stages of the development [5]. Understanding, the genetic diversity and population structure of the parasite is crucial as high genetic diversity especially at the surface- exposed antigens posses great challenge in developing effective malaria vaccine [6]. Almost all *P*. *falciparum* antigens currently under consideration for vaccine development, exhibit polymorphism in the field isolates from various part of the world [7–14]. Furthermore, antibodies induced by parasitic infection are in large part directed against the specific allele. Several studies have demonstrated the presence of B cell epitopes in the repeats of circumsporozoite protein (csp), merozoite surface protein 1 (msp1), merozoite surface protein 2 (msp2) and glutamate rich protien (glurp) of *P*. *falciparum* [15–16]. The immune status of the individuals and exposure to infection play essential role in the clinical response and development of natural immunity to malaria infection, respectively [17]. In endemic areas, immunity to malaria develops slowly and repeated parasite exposure gradually reinforces protective immunity, but disappear within a few months or non-exposure [18]. In addition, if immunity to infection is strain specific, then a state of generalized immunity would develop once exposure had occurred to a large enough sample of the many distinct parasite strains circulating in that region [19]. Therefore, the extensive polymorphisms of surface antigens contribute to the immune evasion and also appear to restrict the effectiveness of vaccine against the *P*.*falciparum* polymorphic proteins. Given the importance of antigenic diversity in influencing the outcome of any vaccine, we have conducted genetic polymorphism of vaccine candidate antigens (*pfmsp1*, *pfmsp2*, *pfglurp*, *pfcsp* and *pfs25*) and antibody responses in *P*. *falciparum* infected individual from Chhattisgarh state, Central India which is second malarious state contributing 12% of malaria cases in India.

## Materials and methods

### Study area, population and sample collection

**Fig 1:**
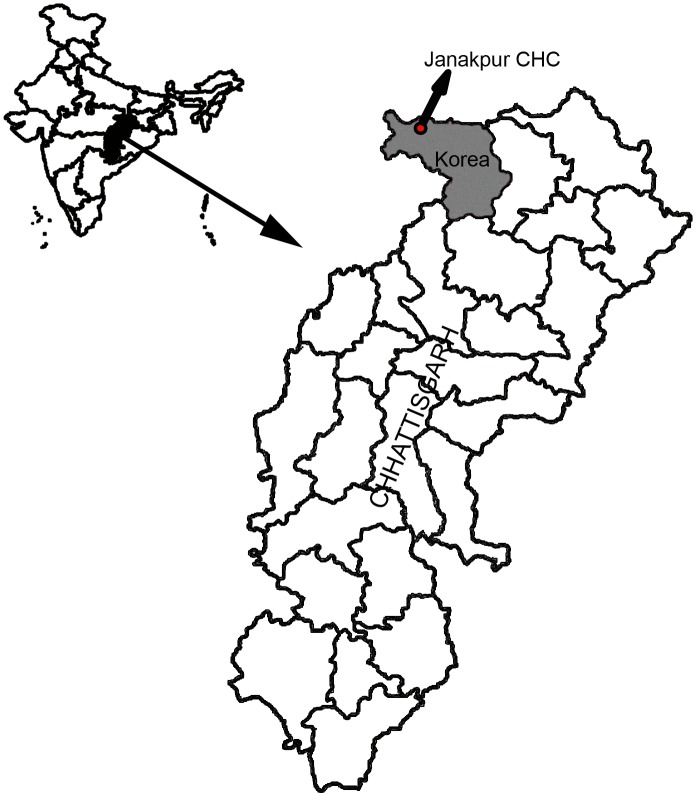
Map of India showing the study site.

This study was carried out at Janakpur Community Health Care (CHC), district Baikunthpur, Chhattisgarh, Central India (Fig 1). This is a secondary health care facility situated in the remote area of the district (23.7191° N, 81.7883° E and 550 M height above sea level) surround by dense forest (60%) and majority of the population is tribal (65%). *P*. *falciparum* is the predominant species followed by *P*. *vivax* and both *Anopheles culicifacies* and *An*. *fluviatilis* are responsible for transmission the disease in this area. A malaria clinic of National Institute for Research in Tribal Health (NIRTH) of Indian Council of Medical Research (ICMR) was established at Janakpur CHC (60 bedded hospital), Baikunthpur district, during August 2013 to March 2015. Symptomatic patients were screened for malaria parasites by microscopy using thick and thin blood smears stained with JSB stain [20]. Patients were given treatment as per National Vector Borne disease control programme [21]. The patient’s socio-demographics and clinical parameters such as age, gender, social groups, clinical history (Headache, Vomiting, Diarrhoea) history of malarial fever, and parasitaemia was recorded on the structured questionnaire. The intravenous blood samples, in sterile conditions, were collected from the patients positive for *P*. *falciparum* malaria after taking written informed consent. Plasma and red blood cells were separated and stored at -80°C for further use in immunological and molecular assays, respectively.

## Ethical approval

The study protocol for patient participation and collection of blood samples for laboratory testing was reviewed and approved by the institutional ethics committee of NIRTH, Jabalpur. All study participants’ provided written informed consent prior to their participation, accroding to ICMR guidelines. A copy of the consent form in the local language was also provided and explained to the patients or parents/Guardian of children. The participation of other institutes was approved by the ICMR, New Delhi, India.

### Genomic DNA extraction and PCR based species identification

Genomic DNA was isolated by QIAamp DNA blood mini kit as per the manufacturer's instructions (Qiagen, CA, USA) and stored at -20°C for further use. Genus and species-specific nested PCR was performed using 18S rRNA gene to detect malaria parasite species (*P*. *falciparum*, *P*. *vivax*, *P*. *ovale*, *and P*. *malariae*), as described previously [22].

### Genotyping of *P*. *falciparum* antigenic markers

The amplification of highly polymorphic antigens; *pfmsp1* (block -2 region), *pfmsp2* (central repeat region), *pfglurp* (polymorphic RII region), *pfcsp* (central repeat region) and *pfs25* was performed in GeneAmp^®^ PCR System 2700 (Applied Biosystems). The PCR reaction mixture and amplification conditions were summarized in S1 Table. Briefly, PCR amplification was carried out in a 25 μL reaction mixture containing 50–100 ng genomic DNA template and 1X of MgCl_2_ free buffer, 1.5 mM of MgCl_2_, 200 μM of dNTPs, 0.2 mM of each primer and 2.5U of Taq polymerase enzyme (Invitrogen Life Sciences, Carlsbad, CA, USA). The appropriate bands excised from the gel and purified using AccuPrep gel extraction kit, according to manufacturer’s protocol (Bioneer Corporation). The purified products were sequenced using both forward and reverse primers, using ABI Big Dye Terminator Ready Reaction Kit Version 3.1 (PE Applied Biosystems, CA, USA) on an ABI-3130xL genetic analyzer. The BioEdit Sequence Alignment Editor and Gene Doc Version 2.6.002 were used to analyze the sequencing electropherograms and generate sequence alignment, respectively. The sequence data generated in this study have been submitted into the NCBI GenBank database. Amino acid sequences of *P*. *falciparum msp1* (block-2), *msp2* (central repeat region), *csp* (central repeat region) from present study and partial/complete amino acid sequences from NCBI database were retrieved using BATCH Entrez and considered for further analysis. Blast clust tool was used to identify the variants from globally available sequence. The details about these isolates have been provided in S2 Table. To study the genetic relationship among the global sequences of *pfmsp*1, *pfmsp*2 and *pfcsp*, Minimal Spanning Tree (MST) was constructed using MLST Clustering algorithm implemented in BioNumerics version 7.6.1 (Applied-Maths, Inc. Austin, TX). The haplotype with the highest numbers of single locus variants (SLVs) was considered as a root haplotype and all other haplotypes as relatives.

### Multiplicity of infection (MOI)

The multiplicity of infection (MOI) was defined as the mean number of *P*. *falciparum* genotypes per infected individual. The MOI was calculated as the proportion of the total number of *P*. *falciparum* genotypes for the same gene and the number of PCR positive isolates.

### Tests of neutrality, recombination and Statistical analysis

Genetic parameters of *P*. *falciparum* gene specific allelic types were determined by different methods implemented MEGA 7 Software [23]. The average number of substitutions (π) per site between any two sequence and heterozygosity per site (θ) was determined as described earlier (Nei and Tamura) [24,25]. Intra and Inter specific transitions and transversion was estimated using the pair-wise differences without any correction. The average number nonsynonymous (dN) and of synonymous substitutions (dS) per site, was estimated using the method of Nei and Gojobori with the Jukes and Cantor [26,27]. Tajima test was performed for detecting the positive natural selection in maintaining genetic polymorphism based on Statistic D and PHI test was performed to detect the overall recombination at each locus the with SPLITSTREE (ver. 4.13) [28]. Window size (W) was set to 100 bases (Φ_w_ = 100), and the statistical significance of Φ_w_ values were assessed using a permutation test with 1,000 iterations {H0 (no recombination, Φ_w_ ≠0) rejected at α = 0.05 in favor of H1 (recombination)} [29].

### Enzyme-linked immunosorbent assay

The total IgG response against the synthetic peptides of pfmsp1 B & T cell epitopes, pfmsp2 B cell epitope, pfcsp B & T cell epitopes and pfglurp were quantified by direct ELISA. Details of peptide sequence were given in the supplementary table (S3 Table). Briefly, 96-well microtitre plates (Nunc-Immuno, Thermo Scientific) were coated overnight at 4°C with 5 μg/ml of each antigenic peptides. After washing, the plates were blocked with 1% skimmed milk for 1 hour at 37°C. Subsequently the plasma samples were diluted (1:100) in PBS-5% skimmed milk, 0.1% Tweeen-20 and incubated for 1 hour at 37°C. The plates were washed four times in PBS 0.1% Tween-20 and incubated for 1 hour at 37°C following the addition of horseradish peroxidase-conjugated goat-antihuman-IgG (1:4000 in PBS-5% skimmed milk, 0.1% Tweeen-20). The assay was developed by adding the tetra-methylbenzidine enzyme substrate and incubated for 30 minutes at 37°C. The reaction was stopped with 2M H_2_SO_4_. The OD was measured at 450 nm using an ELISA plate reader.

To determine the specificity of the assay, sera samples of 16 healthy individuals from non-endemic area (not exposed to malaria) were used as negative control. Prevalence of total IgG immune response for the different antigens were considered positive if their OD values were higher than the mean plus two standard deviations of negative control after subtraction. The percentage prevalence was calculated as follows: (total no. of positive samples / total no. of sample tested) x 100. Odd ratio (OR) was calculated for association between the different age groups.

## Results

### Demographic profiles of the study population

A total of 6718 patients were screened, of which 5.2% (n = 352) were found positive for malaria parasite {*P*.*falciparam* (n = 271), *P*.*vivax* (n = 79) and two mixed infection of *P*.*falciparam* and *P*.*vivax*}. Out of 271, polymerase chain reaction (PCR) positive 180 mono-infected *P*. *falciparam* patients who fulfilled the enrolment criteria were included in the study. The age range across the sample was 01 to 75 years with a mean age of 20.34±15.19 year. Fifty two percent (n = 93) of the patients were female and the rest were male, only 7% (n = 13) of the patients had history of previous malaria infection. Additionally, majority of the participants were from the tribal community (75%, n = 134). Majority (94%) of the patients had fever at the time of enrollment followed by headache (90%), vomiting (75%) and diarrhoea (5%). The mean parasite density was 5827.05 ± 17295(95%CI, 3283.29–8370.80).

### Allelic diversity of *pfmsp1* and *pfmsp2*

**Fig 2:**
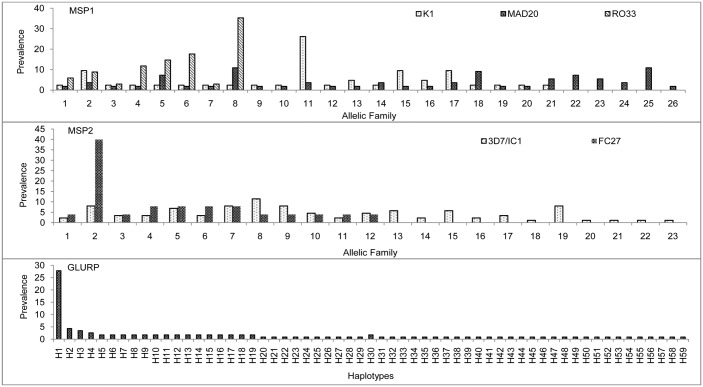
Frequency distribution of different allelic variations of *P*. *falciparum msp1*, *msp2* and *glurp* genes in Indian isolates.

A total 131(73%) samples were successfully sequenced and analyzed for block-2 region of *pfmsp1* gene. The overall allelic prevalence was higher in MAD20 (42%) followed by K1 (32%) and RO33 (26%) (Table 1). Fifty five unique alleles were observed, of which majority were found in MAD20 (26 alleles) followed by K1 (21 alleles) and RO33 (8 alleles) (Fig 2). The average number of amino acid was significantly higher in K1 and MAD 20 when it compared to 3D7 and HN2 reference strains, respectively (Table 1). The different alleles of RO33 were based on only substitutions while in the K1 and MAD20 family both insertion/deletion as well as substitutions were observed. The dN/dS ratio showed the positive selection for K1 and MAD20 alleles while RO33 was found to be neutral (Table 2). Thirty seven percent of the samples were polyclonal infection and the MOI for *msp1* was 1.67. The NCBI GenBank database accession number *msp1* allelic family is KY425824—KY425878.

**Table 1:** Distribution of allelic family and size variations of vaccine candidate genes in Indian *P*. *falciparum* isolates.

Genes (n)	Allelic family type	No. of samples	Percentage	No. of variants	Allele Size	Standard strain (amino acid)	MOI
*pfmsp1* (131)	K1	42	32.06	21	148.48 ± 8.99* (132–171)	3D7 (133)	1.67
	MAD20	55	41.98	26	143.18 ± 8.61* (126–156)	HN2 (127)	
	RO33	34	25.95	8	132.00 ± 0.00 (132–132)	RO33 (132)	
	Mixed	48					
*pfmsp*2 (112)	3D7/IC1	87	77.68	38	210.46 ± 11.69* (193–248)	3D7 (245)	1.28
	FC27	25	22.32	12	191.04 ± 10.86* (184–225)	IGH-CR14 (185)	
	Mixed	31					
*pfglurp* (115)	-	-	-	59	257.04 ± 28.31* (156–317)	3D7 (303)	
*pfcsp* (97)	-	-	-	22	289.30 ± 15.24* (266–321)	3D7 (277)	
*pf*s25 (155)	Wild type	84	54.29	-	155	3D7 (155)	
	A131G	45	29.03	-	155	3D7 (155)	
	V143A	26	16.77	-	155	3D7 (155)	

* P<0.05

**Table 2:** Nucleotide diversity test of neutrality and rate of recombination test of vaccine candidate genes in Indian *P*. *falciparum* isolates.

Gene	No. of Allelic types	S	θ	π	Transitions	Transversions	dN	dS	dN/dS ratio	Tajima's D	PHI test P value
***pfcsp***	22	55	0.02473	0.02143	6.329	6.7402	0.0087	0.0788	0.110	-0.529924	0.000
***pfglurp***	59	21	0.00854	0.00524	0.6166	2.15546	0.0038	0.0072	0.528	-1.209877	0.3584
***pfmsp1*- K1**	21	24	0.01728	0.00899	1.3857	2.0857	0.0103	0.0055	1.873	-1.830012	0.0380
***pfmsp1*- MAD20**	26	38	0.02853	0.03030	5.329	5.246	0.0330	0.0295	1.119	0.233564	0.000
***pfmsp1*- RO33**	8	12	0.01169	0.01380	3.607	1.857	0.0137	0.0149	0.919	0.909062	0.0150
***pfmsp*2-3D7/IC1**	38	53	0.02502	0.033894	9.3058	7.776	0.0415	0.0039	10.641	1.275263	0.000
***pfmsp*2-FC27**	12	59	0.03527	0.03457	7.015	12.136	0.0376	0.0318	1.182	-0.091029	0.0248
***pfs25***	3	2	0.00287	0.00287	0.6666	0.6666	0.0036	0.0000	∞	n/c	

S: Segregating sites; θ: Hetrozygosity per site; π: Average Nucleotide diversity; dN: Average number of non synonymous mutation; dS: Average number of synonymous mutation

Sequencing of the central repeat region of *pfmsp2* gene was successfully analyzed in 112 (62%) samples (Table 1). The predominant alleles was 3D7/ICI (78%) with 38 allelic variant while FC27 was formed among 22% samples with only 12 allelic variants (Fig 2). The amino acid length was significantly lower in both the allelic family when compared to the reference strain (Table 1). The dN/dS ratio indicated the gene under positive selection (Table 2). The transversion for FC27 alleles was much higher as compared to the transitions. Twenty eight percent of the samples were polyclonal infection and the MOI for *pfmsp2* was 1.28. The NCBI GenBank database accession number for *msp2* allelic family is KY425879—KY42928.

### Sequence diversity of *pfglurp*

One hundred fifteen samples were successfully genotyped for *pfglurp* RII repeat region. A total 59 genotypes (Fig 2) were detected with significantly lower average amino acid length when compared to the reference strain 3D7 (Table 1). Tajima test shows that *pfglurp* gene is under negative selection (Table 2). There are 4 type of amino acid repeat sequence unit was present among the all isolates in the well conserved part of RII repeat region with varies in the repeat numbers (Table 3). Apart from these 4 types, another 4 types repeats were also found with minor amino acid substitution in the limited number of isolates (Table 3). However, an extra D or E amino acid was found in between the repeats (S1 Fig). The NCBI GenBank database accession number for *glurp* allelic family is KY425929—KY425987.

**Table 3:** Type of repeats sequence with limited polymorphism of *P*. *falciparum glurp* gene in the Indian isolates.

Type	amino acid repeat sequence	Repeat length (amino acid)	prevalence	average frequency of the repeats ± SD (Min—Max)
1	DKSAHIQHEIVEVEEILPE	19	100	1.01 ± 0.09 (1.00–2.00)
2	DKNEKVEHEIVEVEEILPE	19	100	1.79 ± 0.97 (1.00–4.00)
3	DKNEKGQHEIVEVEEILPE	19	100	4.50 ± 0.91 (2.00–7.00)
4	DKNEKVQHEIVEVEEILPE	19	100	3.14 ± 1.02 (1.00–6.00)
5	DKNEKVNMKIVEVEEILPE	19	2.61	1.00 ± 0.00 (1.00–1.00)
6	DKNEKGQHEIVEGEEILPE	19	1.74	1.00 ± 0.00 (1.00–1.00)
7	DKNEKVQHEIVEVEEVLPE	19	0.89	1.00
8	DKSEKGQHEIVEVEEILPE	19	0.89	1.00
9	DENEKGQHEIVEVEEILPE	19	0.89	1.00

### Diversity in the central repeat region of *pfcsp*

**Fig 3:**
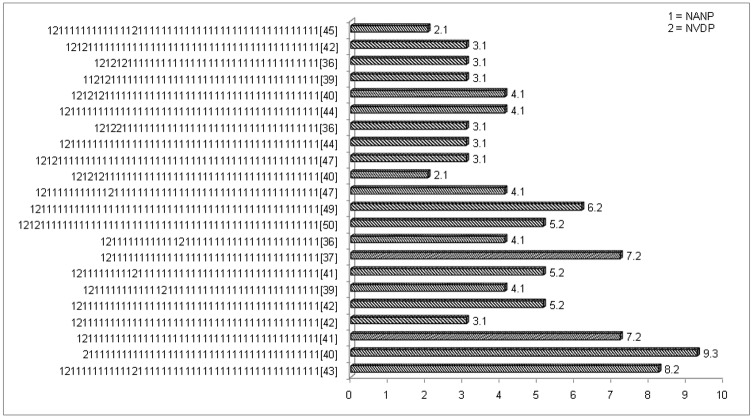
Prevalence of *P*. *falciparum* csp haplotype based on NANP and NVDP repeats and their arrangement.

Twenty two unique haplotypes were observed among 97 samples in the central repeat region (Table 1). The number of tetra-peptide repeats that includes NANP and other minor variants, in this region varied from 36 to 50. Among these haplotypes with most common NANP repeat number was 40 (15%) with 55% samples had tetra-peptide repeats between 40 to 44 numbers. Some repeats contribute three haplotype with same number and some repeats were shared two haplotype however, they had variation in their sequence (Fig 3). GenBank accession number for these haplotypes are KY425988—KY426009

### Sequence diversity in *pfs25*

As expected, the *pfs25* was highly conserved among the 155 successfully sequenced samples and resulted only two amino acid substitution sites (Table 1). Overall, 54% (n = 84) samples were perfectly matched with reference strain 3D7 while only 29% (n = 45) were found substitution at A131G followed by 17% (n = 26) at V143A position. GenBank accession number for these haplotypes are KY426010—KY426012.

### Global analysis of *Pfmsp1*, *Pfmsp2* and *Pfcsp* sequence

**Fig 4:**
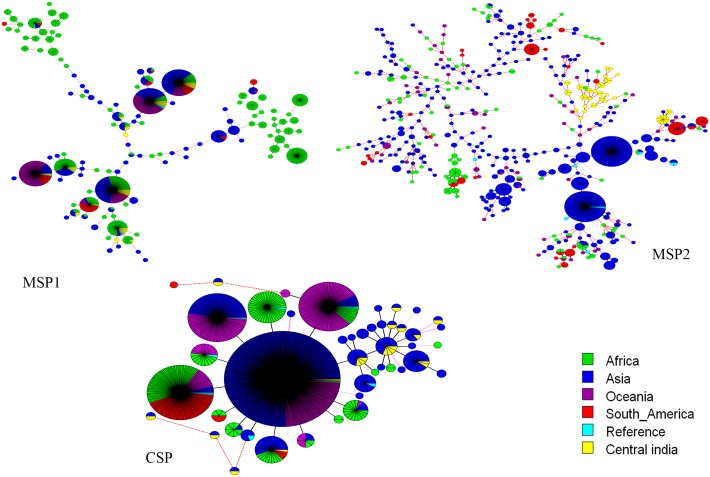
Global population structure of *P*. *falciparum msp1*, *msp2* and *csp* gene. A minimal spanning tree (MST) generated using Bio Numerics software version 7.6.1 showing the relationship from worldwide isolates. Each circle represents and individual haplotype and the size of the circle is proportional to the number of isolates belonging to that haplotypes. The line connecting the circle is branch length.

The minimal spanning tree (MST) analysis of *Pfmsp1* (1149 sequence), *Pfmsp2* (882 sequences) and *Pfcsp* (985 sequence) from global isolates (Africa, Asia, Oceania, South America and reference sequence) were included and used to construct global population structures (Fig 4, S2 Table). The genetic diversity suggested that the African populations show greater diversity as compared to the other continents. The level of genetic diversity in our study population was very similar to the diversity shown by isolates from other Asian Countries.

### Prevalence and levels of total IgG responses against various *P*. *falciparam* antigens / epitopes

**Fig 5:**
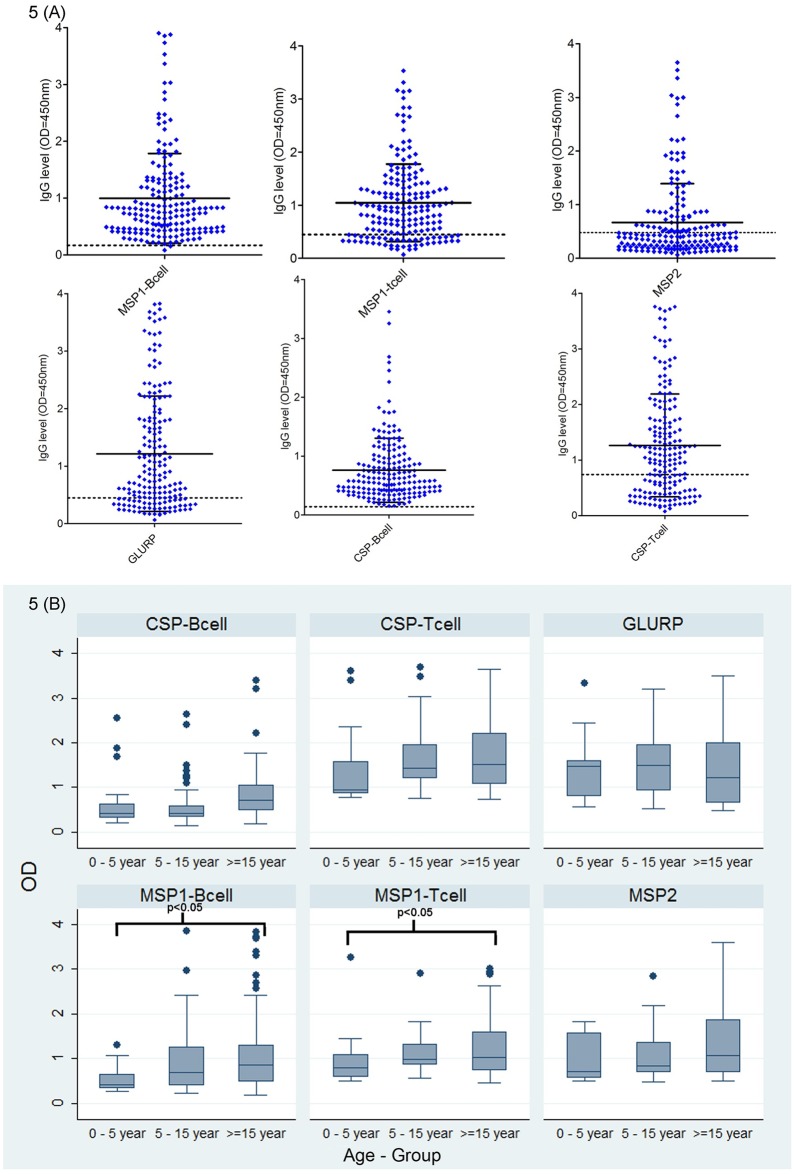
(A) Total IgG antibody levels against synthetic peptide of the *P*. *falciparum* antigens/epitopes. (B) Levels of total IgG antibody responses among different age groups. (Box plots depict median values with 25th and 75th percentile values represented by the bottom and top edges boxes. Small * indicate that the antibody responses statistically significant differences (* p<0.05) when compared among different age groups.

The levels of total IgG was assessed against different antigens/epitope in 180 plasma samples of *P*. *falciparum* infected patients by Enzyme linked Immunosorbent assay. The overall prevalence of total IgG antibody varied greatly between 37% to 97% to different antigens /epitopes (Fig 5A). The higher level of IgG response was found in B-cell epitopes of pfmsp1 (94%) and pfcsp (97%) antigens while significantly lower levels observed against T-cell epitopes of pfmsp1(71%) and pfcsp (62%) antigens. However, only 37% individuals were found positive for B cell epitope of pfmsp2 antigen. In addition, pfglurp antigen showed 64% positivity. The mean antibody levels presented in Fig 5B illustrate significant differences in the IgG levels between the age groups. Furthermore, when assessed the levels of IgG among different age groups, we observed significantly higher levels of antibodies against B-cell epitopes of *pfcsp* and *pfglurp* antigens in the ≥15 years age group than ≤5 year age group (Fig 5B). Further analysis revealed that the IgG antibody response was not associated with the parasite density (S4 Table). Although, samples with low parasite density (500–1000) showed higher antibody response compared to other groups but the difference was not statistically significant (p>0.05). Additionally, the correlation of B-cell epitopes of *pfcsp* response to NANP repeat showed significantly high positive response where the average NANP repeats was ≥42 repeats (p<0.05).

## Discussion

Almost all vaccine candidate antigens present a great challenge for successful development of malaria vaccine due to antigenic diversity in the parasite resulting immune selection in most of the parasite. In this study, we determined the antigenic diversity of sporozoite, merozoite surface proteins, sexual stage antigen and antibody response against these antigens in Indian samples. To the best of our knowledge, this is the first report to analysed genetic polymorphism and antibody responses of five potential vaccine candidates of *P*. *falciparum* (*msp1*, *msp2*, *glurp*, *csp* and *pfs25*) in India. Although, *P*. *falciparum* has been one of the most extensively investigated parasites but there is limited information on genetic diversity of each stage along with antibody response. Present study showed that encoding protein expressed on the surface of the merozoite and sporozoite are more polymorphic than they are expressed during the sexual stage. These results are in agreement with the observation that stage specific surface proteins exhibit high polymorphism when compared with internal antigens [30]. In present study, *P*. *falciparum msp1*, *msp2* and *glurp* were highly diverse from central India region in respect of length as well as sequence motifs with prevalence of all the allelic families of *msp1* and *msp2*, which corroborates with earlier reports on Indian isolates [9,10,13,14,31,32]. Block-2 region of *pfmsp1* exhibit repetitive tri-nucleotides and appears to be subjected to a rapid intra-genic recombination process. Besides these variability, Cavanaugh et.al. (2004) observed that the antibody against block-2 region of *pfmsp*1 are also associated with protection from clinical malaria [33]. The high proportion of multiclonal isolation way is accordance with the previous report [9,10]. One investigator also reported that multiple genotypic infections are associated with malaria severity [34] but in the present study, we have enrolled only uncomplicated malaria. Mono-morphic nature of RO33 family of *msp1* also show many variants in this study as initially it was considered as limited diversified family [35,36]. The sequence polymorphism in K1 and MAD20 types of block-2 looks initially quite extensive but further analysis towards tripeptide motif, it showed limited (5 to 6) subgroups, which was also reported in Tanzanian isolates [37]. The little evidence from field data is available on the selective effects in the malaria vaccine trial and despite of the extensive polymorphism of *pfmsp1*, it is considered as one of the good malaria vaccine candidate genes. Although, the block-2 of *pfmsp1* is considered highly polymorphic but Cavanagh et al 2004 demonstrated that various construct of block 2 region of *pfmsp1* showed good antibody response compared to block 1 region of *pfmsp1*. It was further confirmed that block 2 region showed protective potential of the immunity [33].

*P*. *falciparum msp*2 allelic family are also associated with malaria severity [10] and could be due to super infection of severe cases or strain- specific immunity [38]. It has been demonstrated that anti-msp2 antibodies contribute to the protective immunity [39]. In trial immunization with 3D7 variant of *msp2* prevent re-infection with the parasite carrying alleles of the same family [40]. The asexual blood stage antigens i.e. *pfmsp1*, *pfmsp2* and the ring –infected erythrocyte surface antigen (RESA) are combination vaccine known as combination B, which is immunogenic [41,42] and detected a significant immune response against the central variable region of the *pfmsp2*. In addition, both FC27 and 3D7 family were associated with the extensive polymorphism and immune response. Furthermore, Fluck C et al 2007 demonstrated that family specific dimorphic domain is more likely to account for the *pfmsp2* family specific selective effect [43]. In addition, *P*. *falciparum glurp* has also been considered an important antigen that playing an important role in the induction of protective immunity [44]. Our results suggest that amino acid sequence type 1,2,3,4 (Table 3) was present more frequently in the conserved part R2 region. Although, the antibody response was found only in 64% samples against the peptide of R0 region. Therefore, this 19 mer peptide of R2 region may be considered as future vaccine candidates either in the combination of other stage-specific antigens or multi-allelic vaccine of some antigens. This finding is consistent with the previous studies [13,44]. R2 repeat region is the immunodominant region and targeted by the human IgG antibody, which is effective in antibody-dependent cellular inhibition (ADCI).

*P*. *falciparum* csp antigen is a part of RTS’S vaccine, which showed only partial protection (50%) in the phase- III trial [45], and no clear reason behind the partial efficacy was established. In this study, we observed high degree of amino acid repeats polymorphism in the central repeat region where almost 54% of the samples contained 40 to 44 tetrapeptide (NANP) repeats, which is consistent with our previous findings and other studies [12,46]. In addition to NANP repeats, other minor repeats such as NVDP, NADP and NVNP were also found in the present study, which may be influence the antibody response against *pfcsp*. A large number of haplotypes observed in the present study is consistent with this region of the circumsporozoite gene being under diversifying selection. The selection of non-vaccine variant of CSP following immunogenicity with RTS, S/AS01 vaccine, which is CSP based was not allele specific [47]. Therefore, this study makes an important understanding the type and distribution of naturally occurring polymorphism in RTS, S vaccine candidate antigens in a population from Chhattisgarh-Central India. Furthermore, as expected, sexual stage antigen *pfs25* was found to be conserved and only two site mutated one is Alanine with Glycine and other Valine with Alanine, which is in agreement with previous study [12].

In the present study, agreement with large parasite diversity in these genes may be due to higher malaria transmission intensity as the extent of polymorphism may vary with transmission intensity by applying inter-clonal parasite mating and intragenic recombination [48]. Other factors such as frequent migration within sub-region, gametocyte reservoir and antimalarial drug resistance may have intensified the level of *P*. *falciparum* diversity. Therefore, the understanding the population structure and diversity of *P*. *falciparum* has implication for vaccine, drug and epidemiological studies including monitoring of malaria [49,50].

The blood stage (msp1, msp2 and glurp) and liver stage (csp) vaccine candidates have been reported to have B cell and T cell recognition domain. In the present investigation, we determined the IgG antibody response against synthetic peptides representing the conserved region of B cell & T cell of msp1, csp and B cell of msp2, glurp among patients infected with *P*. *falciparum* in Central India, where malaria is endemic [51]. In this study, majority of the plasma samples reacted against the B cell epitope of pfmsp1 and pfcsp despite its short amino acid sequence. In addition, the level of IgG antibody responses varies in different antigens/epitopes, which is in agreement with the previous study where T cell response was significantly lower than B-cell response, suggesting that the peptide is immunogenic [52]. However, several other studies have found difference in the immune response to these antigens may be due the different structure of the epitopes [53]. The existence of allele specific antibodies in *P*. *falciparum* infected samples has been demonstrated, however their role in protection was not established [52]. Furthermore, a negative association between antibody response to pfmsp2 and risk of malaria was observed, suggesting a possible protective role for anti-msp2 antibody in natural infection [54]. Although we found a low level of antibody response against pfmsp2. Several studies have demonstrated that antibodies against both repeat and non-repeat region of glurp can inhibit parasite growth in-vitro [55]. In the present study we also observed higher level of IgG antibody response against glurp antigen, suggest partial protective response and it should be considered as future vaccine candidate antigen. The age specific antibody levels against B-cell epitopes of pfcsp and pfglurp antigens showed a positive association with increasing age suggesting the role of intrinsic age-related factors in immune maturation. The world wide dynamics of *P*. *falciparum* populations is complex and distribution of different parasite strains differ from region to region and evolves over time possibly because of immune selection. Therefore, a study from a range of different settings are required to show how the vaccine candidates dependent to the different alleles of the vaccine candidate antigens.

The strengths of this study is the comprehensive use of sensitive and standardized assays, rendering our findings amenable to a detailed analysis of genetic polymorphisms as well as antibody response in *P*. *falciparum* vaccine candidate antigens. In the present study, we aimed for such analysis to be relevant for epidemiologic investigations, malaria transmission dynamics and vaccine design.

The limitation of present study is that we could not assess the genetic polymorphism of *P*. *falciparum* csp T-cell (Th2 and Th3) epitopes. However, the present study has robustly genotypes the *P*. *falciparum* merozoite, sporozoite surface and sexual stage protein to obtain a conclusive genetic diversity data set for this region. It has been proposed that diversity in the repeat region might have evolved as an immune evasion mechanism by the parasite in which the host mount the protective immune response against this region while the parasite escapes into the liver [56].

## Conclusion

This is a first extensive study that contributes in understanding the type and distribution of naturally evolved genetic polymorphism in the *P*. *falciparum* vaccine candidate antigens from Chhattisgarh, Central India, where malaria is endemic. There is probably some association between malaria hyper endemicity and the parasite mixture as well as the number of alleles present in the area. This data would be helpful in the future malaria vaccine trial in India and to monitor changes in the parasite population. Furthermore, the characterization of the pattern of antibody response to malarial antigens/epitopes may also be useful in monitoring the effect of malaria vaccine. The simultaneous presence of antibody response, in our population, to more than one antigen is indicative of a lower frequency of malarial infection and suggests that a successful malaria vaccine should contain multiple antigens. Therefore, similar study should be carried out on a large scale to cover the entire country to map the different endemic regions. Finally, the global analysis of allelic variants reported in this study would be helpful in the identifying the predominant allele in the region and may aid in designing region specific vaccine and would pave the way for an effective malaria intervention measures.

## Supporting information

S1 Table Primer sequence and PCR condition used for amplification of *P*. *falciparum* genes. (DOCX)

S2 Table The available accession numbers, country, continent and references used in this study. (XLSX)

S3 Table Detail sequences of synthetic peptides used in the study. (DOCX)

S4 Table IgG antibody response against different level of parasite density. (DOCX)

S1 Fig Amino acid sequence diversity in RII repeat region in *glurp*. (TIF)
