# Supplementary material for: Genetic diversity and antibody responses against Plasmodium falciparum vaccine candidate genes from Chhattisgarh, Central India: Implication for vaccine development
Source: PLoS One. 2017 Aug 7;12(8):e0182674. doi: 10.1371/journal.pone.0182674 (PMC5546615; doi:10.1371/journal.pone.0182674)
Supplement: S1 Table — (DOCX) [file pone.0182674.s001.docx]

**Table S1: Primer Sequence and PCR condition used for amplification of *P.falciparum* genes**

| **Gene** | **Primer name** | **Primer Sequence** | **PCR product**  **Length (bp)** | **Denaturation** | **Annealing** | **Elongation** | **No of**  **Cycles** |
| --- | --- | --- | --- | --- | --- | --- | --- |
| *pfmsp-1* Primary | MSP1A | CACAATGTGTAACACATGAAAG | 646 bp | 94 °C, 1 min | 55 °C, 1 min | 72 °C, 1 min | 35 |
|  | MSP1B | AGTACGTCTAATTCATTTGCAC | 646 bp | 94 °C, 1 min | 55 °C, 1 min | 72 °C, 1 min |  |
| *pfmsp-1* Nested | MSP1C | TAGAAGCTTTAGAAGATGCAG | 555 bp | 94 °C, 1 min | 53 °C, 1 min | 72 °C, 1 min | 30 |
|  | MSP1D | GACAATAATCATTAGCACATAC | 555 bp | 94 °C, 1 min | 53 °C, 1 min | 72 °C, 1 min |  |
| *pfglurp* | GLPF | TGCAAGTGTTGATCCTGAAGT | 1063 bp | 94 °C, 1 min | 55 °C, 1 min | 72 °C, 1.3 min | 35 |
|  | GLPR | AATGTAGGTACCACGGGTTC | 1064 bp | 94 °C, 1 min | 55 °C, 1 min | 72 °C, 1.3 min |  |
| *pfcsp* | CSP1 | TTAGCTATTTTATCTGTTTCTTCC | 1177 bp | 94 °C, 1 min | 51 °C, 1 min | 72 °C, 1.3 min | 35 |
|  | CSP2 | TAAGGAACAAGAAGGATAATACC | 1177 bp | 94 °C, 1 min | 51 °C, 1 min | 72 °C, 1.3 min |  |
| *pfs25* Primary | P251F | ATGAATAAACTTTACAGTTTGT | 580 bp | 94 °C, 1 min | 48 °C, 1 min | 72 °C, 1 min | 35 |
|  | P251R | CAGTACATATAGAGCTTTCAT | 580 bp | 94 °C, 1 min | 48 °C, 1 min | 72 °C, 1 min |  |
| *pfs25* Nested | N251F | AGTTACCGTGGATACTGTAT | 512 bp | 94 °C, 1 min | 51 °C, 1 min | 72 °C, 1 min | 30 |
|  | P251R | CAGTACATATAGAGCTTTCAT | 512 bp | 94 °C, 1 min | 51 °C, 1 min | 72 °C, 1 min |  |
| *pfmsp-2* Primary | Msp2A | ATGAAGGTAATTAAAACATTGTC | 760 bp | 94 °C, 1 min | 53 °C, 1 min | 72 °C, 1 min | 35 |
|  | Msp2B | TTATTGAAGCAATATTACTAGAG | 760 bp | 94 °C, 1 min | 53 °C, 1 min | 72 °C, 1 min |  |
| *pfmsp-2* Nested | Msp2C | AGCAACACATTCATAAACAATG | 750 bp | 94 °C, 1 min | 54 °C, 1 min | 72 °C, 1 min | 30 |
|  | Msp2D | CACAGTTTTCTTTGTTACCATC | 750 bp | 94 °C, 1 min | 54 °C, 1 min | 72 °C, 1 min |  |
