# Supplementary material for: Genetic diversity and antibody responses against Plasmodium falciparum vaccine candidate genes from Chhattisgarh, Central India: Implication for vaccine development
Source: PLoS One. 2017 Aug 7;12(8):e0182674. doi: 10.1371/journal.pone.0182674 (PMC5546615; doi:10.1371/journal.pone.0182674)
Supplement: S3 Table — (DOCX) [file pone.0182674.s003.docx]

**Table S3: Detail sequences of synthetic peptides used in the study.**

| **S. No** | **Name of Antigens/epitopes** | **Peptide sequences** |
| --- | --- | --- |
| 1 | pfmsp1:Bcell | NSGCFRHLDEREECKCLL |
| 2 | pfmsp1:Tcell | LKPLAGVYRSLKKQIEK |
| 3 | pfmsp2:Bcell | ETESYKQLVAKLDKLEALVV |
| 4 | pfglurp | QSEKSLVSENVPSGLDID |
| 5 | pfcsp:Bcell | NANP NANPNANP |
| 6 | pfcsp:TCell | DIEKKICKMEKCSSVFNVVNS |
