# Supplementary material for: Genetic diversity and antibody responses against Plasmodium falciparum vaccine candidate genes from Chhattisgarh, Central India: Implication for vaccine development
Source: PLoS One. 2017 Aug 7;12(8):e0182674. doi: 10.1371/journal.pone.0182674 (PMC5546615; doi:10.1371/journal.pone.0182674)
Supplement: S4 Table — (DOCX) [file pone.0182674.s004.docx]

**Table S4: IgG Antibody response against different level of parasite density.**

| **Parasite density** | **CSP-Bcell**  **%**  **(95% CI)** | **CSP-Tcell**  **%**  **(95% CI)** | **GLURP**  **%**  **(95% CI)** | **MSP1-Bcell**  **%**  **(95% CI)** | **MSP1-Tcell**  **%**  **(95% CI)** | **MSP2**  **%**  **(95% CI)** |
| --- | --- | --- | --- | --- | --- | --- |
| **< 100**  **(n= 14)** | 100.0 | 64.3  (35.1 - 87.2) | 64.3  (35.1 - 87.2) | 100.0 | 92.9  (66.1 - 99.8) | 35.7  (12.8 - 64.9) |
| **100 – 250**  **(n= 16)** | 100.0 | 81.3  (54.4 - 96.0) | 56.3  (29.9 - 80.2) | 87.5  (61.7 - 98.4) | 87.5  (61.7 - 98.4) | 37.5  (15.2 - 64.6) |
| **250 – 500**  **(n= 23)** | 100.0 | 65.2  (42.7 - 83.6) | 82.6  (61.2 - 95.0) | 95.7  (78.1 - 99.9) | 73.9  (51.6 - 89.8) | 30.4  (13.2 - 52.9) |
| **500 – 1000**  **(n= 31)** | 93.6  (78.6 - 99.2) | 74.2  (55.4 - 88.1) | 71.0  (52.0 - 85.8) | 96.8  (83.3 - 99.9) | 80.7  (62.5 - 92.5) | 45.2  (27.3 - 64.0) |
| **1000 – 2500**  **(n= 41)** | 100.0 | 53.7  (37.4 - 69.3) | 56.1  (39.7 - 71.5) | 95.1  (83.5 - 99.4) | 58.5  (42.1 - 73.7) | 41.5  (26.3 - 57.9) |
| **2500 – 5000**  **(n= 11)** | 100.0 | 36.4  (10.9 - 69.2) | 54.6  (23.4 - 83.3) | 100.0 | 72.7  (39.0 - 94.0) | 36.4  (10.9 - 69.2) |
| **5000 – 10000**  **(n= 12)** | 100.0 | 75.0  (42.8 - 94.5) | 75.0  (42.8 - 94.5) | 83.3  (51.6 - 97.9) | 66.7  (34.9 - 90.1) | 33.3  (9.9 - 65.1) |
| **>10000**  **(n= 32)** | 87.5  (71.0 - 96.5) | 50.0  (31.9 - 68.1) | 56.3  (37.7 - 73.6) | 93.8  (79.2 - 99.2) | 56.3  (37.7 - 73.6) | 28.1  (13.7 - 46.7) |
