# Supplementary figures and images for: Genetic diversity and antibody responses against Plasmodium falciparum vaccine candidate genes from Chhattisgarh, Central India: Implication for vaccine development
Source: PLoS One. 2017 Aug 7;12(8):e0182674. doi: 10.1371/journal.pone.0182674 (PMC5546615; doi:10.1371/journal.pone.0182674)

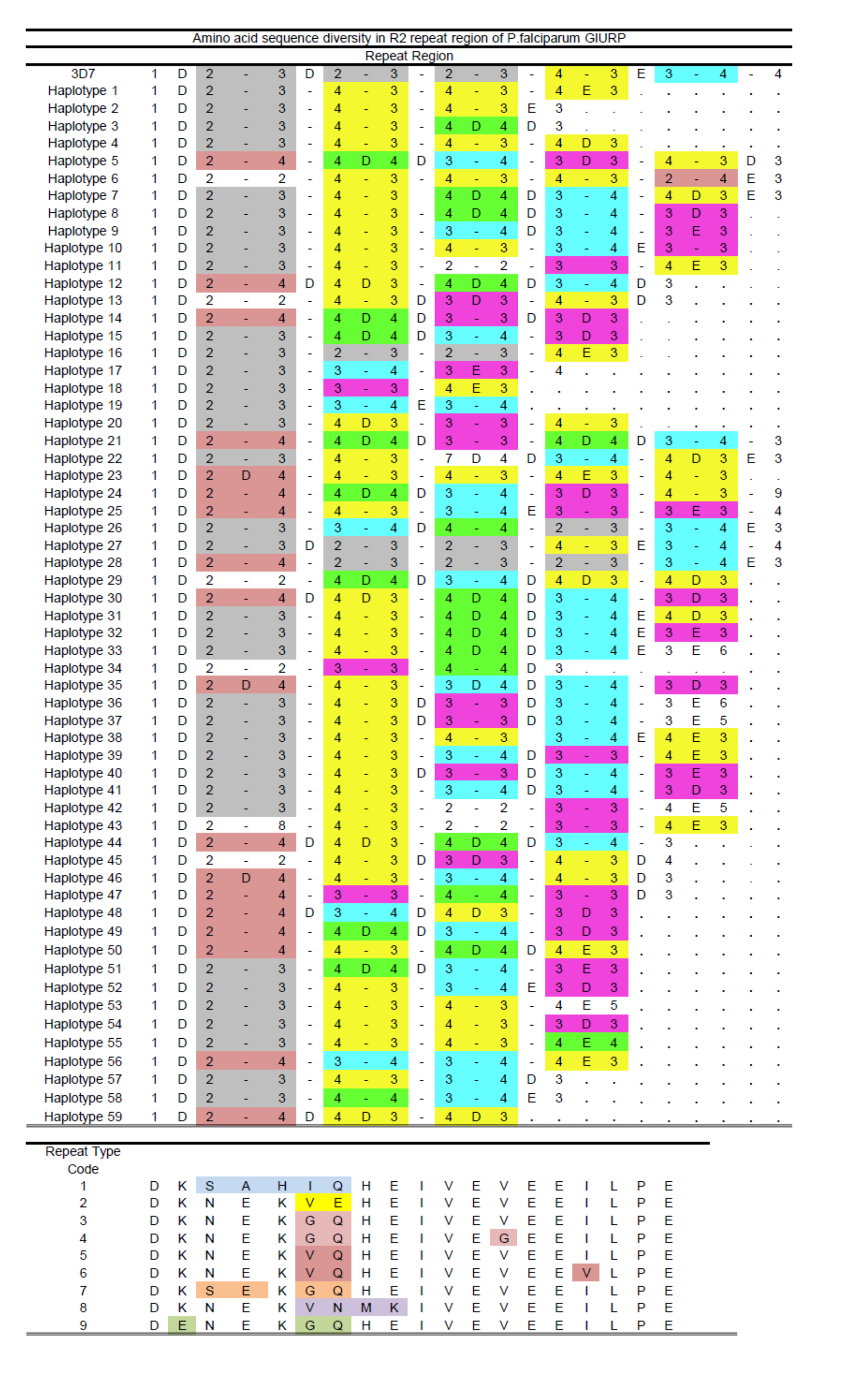

Supplement: S1 Fig — (TIF) [file pone.0182674.s005.tif]
